# New insights into mitochondrial quality control in anthracycline-induced cardiotoxicity: molecular mechanisms, therapeutic targets, and natural products

**DOI:** 10.7150/ijbs.103810

**Published:** 2025-01-01

**Authors:** Weili Li, Yuqin Zhang, Yan Wei, Guanjing Ling, Yawen Zhang, Yilin Li, Shujuan Guo, Nannan Tan, Lin Ma, Wei Li, Qianbin Sun, Wei Wang, Yong Wang

**Affiliations:** 1 School of Traditional Chinese Medicine, Beijing University of Chinese Medicine, Beijing 100029, China.; 2 Dongzhimen Hospital, Beijing University of Chinese Medicine, Beijing 100029, China.; 3 Guangzhou University of Chinese Medicine, Guangzhou 510006, China.; 4 Beijing Key Laboratory of TCM Syndrome and Formula, Beijing University of Chinese Medicine, Beijing 100029, China.; 5 Key Laboratory of TCM Syndrome and Formula (Beijing University of Chinese Medicine), Ministry of Education, Beijing 100029, China.; 6 Anhui University of Traditional Chinese Medicine, Anhui 230012, China.

**Keywords:** anthracycline, cardiotoxicity, mitochondrial quality control, mitophagy, mitochondrial dynamics, mitochondrial biogenesis, natural products

## Abstract

Anthracyclines (ANTs) are widely used in cancer therapy, particularly for lymphoma, sarcoma, breast cancer, and childhood leukemia, and have become the cornerstone of chemotherapy for various malignancies. However, it is associated with fatal and dose-dependent cardiovascular complications, especially cardiotoxicity. Mitochondrial quality control mechanisms, encompassing mitophagy, mitochondrial dynamics, and mitochondrial biogenesis, maintain mitochondrial homeostasis in the cardiovascular system. Recent studies have highlighted that mitochondrial quality control mechanisms play considerable roles in ANTs-induced cardiotoxicity (AIC). In addition, natural products targeting mitochondrial quality control mechanisms have emerged as potential therapeutic strategies to alleviate AIC. This review summarizes the types, incidence, prevention, treatment, and pathomechanism of AIC, delves into the molecular mechanisms of mitochondrial quality control in the pathogenesis of AIC, and explores natural products that target these mechanisms, so as to provide potential targets and therapeutic drugs for address the clinical challenges in AIC prevention and treatment, where no effective medicines are available.

## 1. Overview of AIC

### 1.1 Types of AIC

The discovery of the first anthracycline compound daunorubicin from *Streptomyces peucetius* in the 1950s was a major breakthrough in the realm of oncology 1, and thousands of daunorubicin and doxorubicin (DOX) analogues were subsequently produced, fostering a new era in cancer therapeutics 2. Nowadays, the most clinically pertinent anthracyclines (ANTs), notably DOX, daunorubicin, darubicin, and epirubicin, have emerged as the cornerstone of chemotherapy for an extensive spectrum of malignancies, encompassing lymphoma, sarcoma, breast cancer, and childhood leukemia, among others 3. While ANTs have undeniably improved long-term survival rates among cancer patients, their efficacy comes at the expense of severe, dose-dependent cardiovascular complications, particularly cardiotoxicity 4. Anthracycline-induced cardiotoxicity (AIC) includes a broad spectrum of cardiac injury resulting from ANTs exposure, ranging from subclinical cardiomyocyte injury to heart failure (HF). According to the time of onset, AIC was categorized into three types 5-9: (1) Acute AIC, which occurred within 2 weeks after the end of a single dose or course of ANTs treatment and manifested as supraventricular arrhythmia, transient cardiac dysfunction, and alterations in electrocardiogram. (2) Early-onset Chronic AIC, with onset within 1 year. It is the most prevalent clinically type of cardiotoxicity and usually manifests as dilated and hypokinetic cardiomyopathy, often progressing to HF. (3) Delayed-onset Chronic AIC, occurring years or even decades following chemotherapy, this form of AIC is characterized by the clinical manifestations of HF, cardiomyopathy, and arrhythmia, underscoring the long-term impact of anthracyclines on cardiac health.

### 1.2 Incidence of AIC

The incidence of AIC varied according to different definitions. Specifically, when cardiotoxicity was defined as clinical congestive HF (CHF), the incidence of DOX at 400, 550, and 700 mg/m^2^ was 3%, 7%, and 18%, respectively 10. Subsequent investigations and analyses further corroborated this trend, revealing an escalation in CHF incidence to 4.7%, 26%, and 48% at the same dosage levels 11. Notably, the incidence of clinically asymptomatic cardiotoxicity far surpassed that of symptomatic CHF, with a decline in absolute value ≥ 10% in left ventricular ejection fraction (LVEF) from baseline and to below 50% occurring in 9%, 18%, 38%, and 65% of patients receiving DOX doses of 250 mg/m^2^, 350 mg/m^2^, 450 mg/m^2^, and 550 mg/m^2^, respectively 11. These early retrospective studies resulted in a lifetime cumulative exposure of DOX limit to less than 450 mg/m^2^ in patients not currently receiving thoracic radiotherapy. In a contemporary prospective study involving 2625 patients receiving ANTs-based chemotherapy, cardiotoxicity (defined as echocardiographic decrease in LVEF ≥ 10% to absolute value less than 50%) occurred in 9% of patients 9. The use of cardiac biomarkers, such as troponin I, reduces the incidence of cardiotoxicity by detecting early myocardial changes before the reduction in LVEF occurring. Among patients receiving high-dose chemotherapy, predominantly ANTs, a staggering 30% exhibited elevated troponin I levels within 72 hours post-chemotherapy cycle, which is highly predictive of future left ventricular dysfunction 12. Nevertheless, the susceptibility of different individuals to cardiotoxicity becomes difficult to predict due to genetic variability and the differentiation of cardiovascular risk factors 13.

### 1.3 Risk stratification of AIC

In contemporary oncology clinical practice, it is highly recommended to conduct a comprehensive cardiovascular toxicity risk assessment prior to initiating potentially cardiotoxic antitumor therapies. This decision-making process must weigh the benefits against the risks associated with treatment, ultimately aiming to not only mitigate the incidence and mortality rates of cardiovascular diseases, but also reduce the interruption of cancer treatment caused by cardiovascular events 14. Risk factors for cardiovascular toxicity mainly include history of heart disease, abnormal baseline of myocardial biomarkers, underlying diseases, age, and lifestyle 15. Notably, the Heart Failure Association of the European Society of Cardiology Cardio-Oncology Study Group and International Cardio-Oncology Society jointly released the HFA-ICOS cardiotoxicity risk score, which provides a risk stratification tool for oncologists, hematology oncologists and cardiologists to evaluate the cardiovascular toxicity caused by different chemotherapy drugs 16. The HFA-ICOS cardiotoxicity risk score encapsulates baseline risk stratification before the receipt of the following cancer treatment: Anthracycline-based chemotherapy, Human epidermal growth factor receptor-2 (HER-2) targeted therapy, Vascular endothelial growth factor inhibitors, Targeted kinase inhibitors, Proteasome inhibitors, Immunomodulatory drugs, Rapidly accelerated fibrosarcoma (RAF) and mitogen-activated extracellular signal-regulated kinase (MEK) inhibitors, and androgen-deprivation therapy. However, the HFA-ICOS cardiovascular risk assessment tool also has limitations. For example, the existing known risk factors cannot fully explain the interindividual differences in susceptibility to cardiovascular toxicity, hinting at genetic susceptibility as an emerging potential risk factor. Wang *et al.* have found that roundabout guidance receptor 2 (ROBO2) is a novel susceptibility gene for pediatric patients with cardiovascular toxicity, which promotes cardiac fibrosis through the Slit-Robo-Smad pathway, leads to disordered remodeling of extracellular matrix and exacerbates heart failure, suggesting that genetic factors will be an important basis for risk stratification in the future 17. Therefore, further clinical studies should incorporate the following elements to more accurately detect the risk of cancer treatment exposure: 1) sophisticated cardiovascular imaging algorithms; 2) apply cutting-edge multi-omics techniques to establish genetic markers of potential risk associated with cancer therapy exposure; And 3) utilize an *in vitro* bioplatform of induced pluripotent stem cells to reimage cardiomyocytes from cancer patients and test their susceptibility to certain cancer therapies.

### 1.4 Prevention and treatment of AIC

Two primary prevention approaches for AIC are: 1) to reduce cardiotoxicity by continuous infusion, or liposome capsule administration. 2) Concomitant use of cardioprotective agent (e.g., dexrazoxane (DXZ)). Secondary prevention strategies include angiotensin converting enzyme inhibitors (ACEI), angiotensin receptor blocker (ARB), angiotensin receptor neprilysin inhibitors (ARNI), β-blockers, and statin. Primary treatment drugs for AIC includes DXZ, ACEI, ARB, ARNI, β-blockers and sodium-dependent glucose transporters 2 (SGLT-2) inhibitors.

Liposome encapsulation alters pharmacokinetics and tissue distribution without compromising antitumor efficacy 18. Two types of liposomal DOX are employed clinically: pegylated or unpegylated, with pegylated being more widely used in the United States. Pegylation involves the covalent attachment of surface-bound methoxy polyethylene glycol to the liposomal phospholipid bilayer. This prevents the liposome from being engulfed by the monocyte-macrophage system, reducing its antigenicity and prolonging its half-life 19. However, the number of clinical studies is small, and data on long-term follow-up are lacking, which may have contributed to the limited widespread clinical application of liposomal DOX.

DXZ is the only cardioprotective agent approved by the Food and Drug Administration (FDA) for AIC 20. It is a potent cardioprotective agent against ANTs in a variety of cancer types in children and adults 21, 22. The ability of DXZ to chelate iron is thought to be a major mechanism of myocardial protection 23. However, other iron chelators such as deferasirox are not cardioprotective 24. Lyu *et al.* demonstrated that DXZ exerted cardioprotection by tightly binding to the adenosine triphosphate (ATP)-binding site of topoisomerase II (Top II), thereby preventing ANTs from binding to the Top II complex 25. Some studies have pointed out that DXZ may interfere with the antitumor efficacy of ANTs 26, but which have not been replicated in other clinical trials, and animal studies suggest that DXZ and ANTs have synergistic anticancer effects 27, 28. Another controversy about DXZ is the potential risk of increasing the occurrence of secondary malignant tumors 29, but which has not been found in several subsequent studies 30, 31.

ACEI/ARB/ARNI or β-blocker has not been widely used due to lack of clinical evidence. Several clinical studies have investigated ACEI/ARB/ARNI or β-blockers for the prevention and treatment of AIC. A clinical trial in breast cancer patients found that candesartan did not prevent LVEF reduction but was associated with a small reduction in left ventricular end-diastolic volume and preservation of global longitudinal strain 32. A study involving breast cancer patients receiving ANTs-based chemotherapy found that carvedilol had no effect on the incidence of early LVEF reduction but resulted in a significant reduction in troponin levels and a reduction in the incidence of diastolic dysfunction 33. In the latest trial, the combination of candesartan and carvedilol had no significant protective effect on LVEF in patients at high risk for cardiotoxicity from ANTs-based chemotherapy 34.

SGLT-2 inhibitors were the cornerstone drugs for the treatment of chronic HF. Recent studies have shown that SGLT-2 inhibitor empagliflozin reduced the inflammation, fibrosis, ferroptosis and apoptosis of myocardial cells related to ANTs chemotherapy, and prevented the decline of cardiac function 35, 36. A retrospective clinical study has suggested that the incidence of cardiac events was reduced in 3033 cancer patients treated with ANTs combined with SGLT-2 inhibitors 37. Therefore, SGLT-2 inhibitors have been added as cardioprotective agents for AIC, but it has not been widely applied due to insufficient clinical evidence.

### 1.5 Molecular mechanisms of AIC

**Figure 1:**
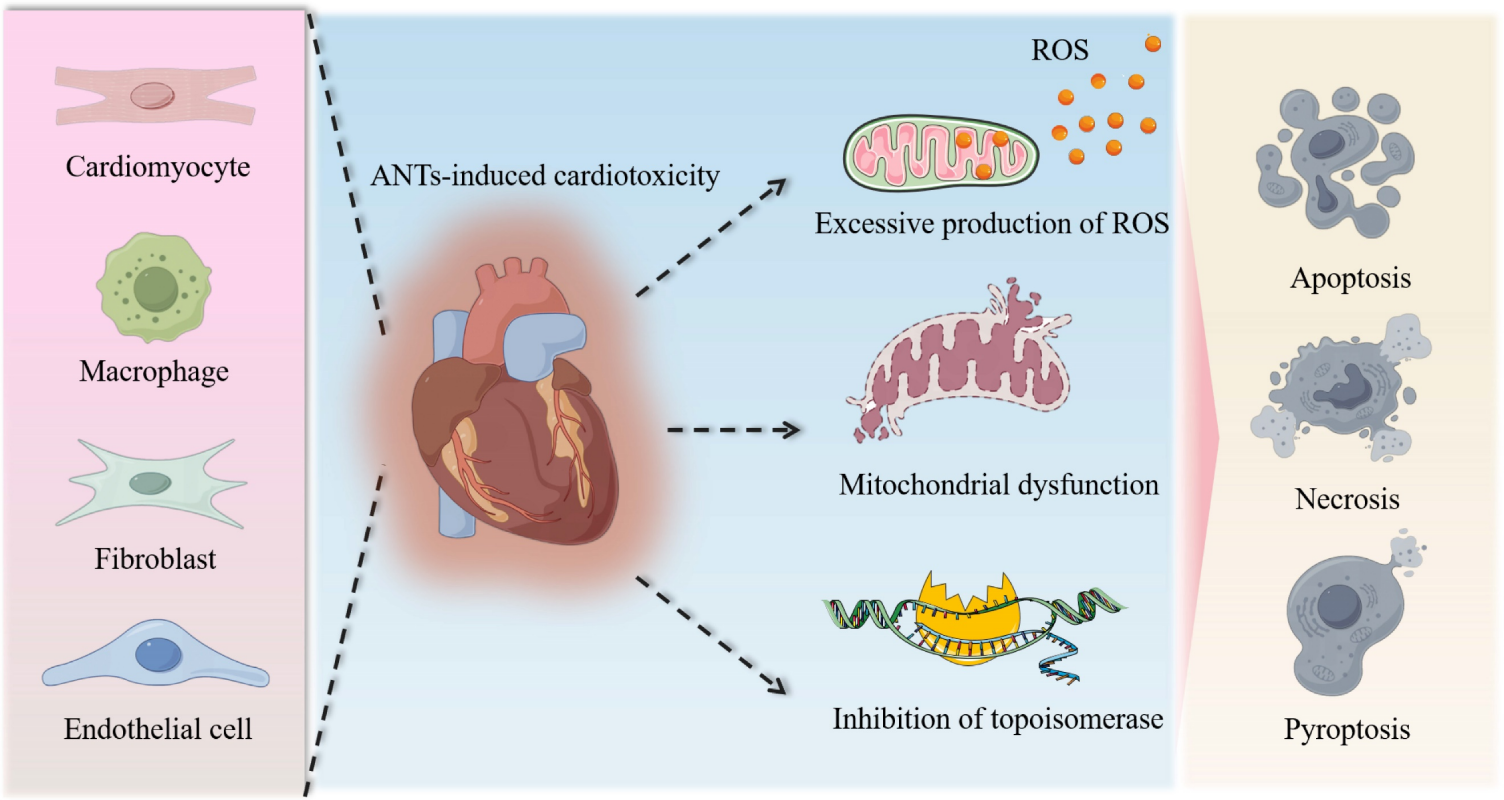
** Molecular mechanisms of AIC.** The intricate pathological mechanisms underlying AIC encompass extensive damage to diverse cardiac cell types, notably cardiomyocytes, macrophages, fibroblasts, and endothelial cells, among others. This cellular injury is characterized by the accumulation of reactive oxygen species, the impairment of mitochondria, and the inhibition of topoisomerases, collectively culminating in cell death, including apoptosis, necrosis, and pyroptosis.

The molecular mechanisms of AIC have been proposed to include reactive oxygen species (ROS) production, topoisomerase inhibition, mitochondrial dysfunction, and multiple forms of cell death (Figure 1). Among them, the most widely accepted hypothesis is the generation of excess ROS through electron exchange between the anthraquinone and cellular electron donors 38, 39. ANTs also form complexes with iron that undergo redox cycles and generate oxygen free radicals 40. Although *in vivo* and *in vitro* studies have confirmed an increase in intracellular ROS production after ANTs treatment, neither antioxidants nor iron chelators can prevent cardiomyopathy 24, 41, 42.

ANTs are mitochondrial toxin that target and accumulate in mitochondria 43. This accumulation is largely attributed to the cationic nature of ANTs, which have high affinity for cardiolipin, a negatively charged major phospholipid component locating in the inner mitochondrial membrane. The binding of ANTs to cardiolipin results in the formation of an irreversible complex that is retained in the mitochondrial inner membrane 44, 45. Proteins of the electron transport chain need to bind to cardiolipin to function properly, and more superoxide is produced due to disruption of the cardiolipin protein interface by ANTs, resulting in dysfunction of the mitochondrial respiratory chain. In addition, other membrane proteins, such as those responsible for carnitine transfer, can also be adversely affected by ANTs, leading to decrease in mitochondrial function 46. ANTs also significantly reduced the oxidation of long-chain fatty acids, increased glucose metabolism, and comprehensively changed the metabolic state from aerobic to anaerobic metabolism 47. Some studies have also reported that DOX treatment affects myocardial mitochondrial gene expression, leading to defects in the respiratory chain/oxidative phosphorylation system of myocardial mitochondria and reduced ATP production 48. The above damage to mitochondrial function eventually leads to pathological changes in mitochondrial ultrastructure 49. The formation of ROS induced by ANTs forms a vicious circle with defects in the respiratory chain/oxidative phosphorylation system, reduced ATP production, and conversion of metabolic substrate utilization, which aggravate mitochondrial damage and bioenergy crisis, and eventually lead to myocardial cell death. Considering that mitochondria are extremely abundant in cardiomyocytes and that the heart has lower levels of antioxidant enzymes such as catalase and superoxide dismutase (SOD) than other organs 50, it is reasonable that the heart is more sensitive to the ROS production induced by ANTs.

Top II unwinds deoxyribonucleic acid (DNA) strands during DNA replication, transcription or recombination 51. There are two types of Top II: Top IIα and Top IIβ. Top IIα, mainly found in proliferating cells, is required for DNA replication, and is thought to be the molecular basis for the tumor-killing activity of ANTs 52. In contrast, Top IIβ is present in all resting cells, including cardiomyocytes. Top IIβ has recently been identified as a key mediator of AIC 53. ANTs disrupt the normal catalytic cycle of Top IIβ and causes DNA double-strand breaks, which further changes the transcriptome and leads to defects in mitochondrial biogenesis and increased ROS, as well as disarrangement and vacuolization of myofibrils in cardiomyocytes 54. Among them, Top 2β and ANTs significantly reduce the expression of peroxisome proliferator-activated receptor-γ coactivator 1-α (PGC-1α) and peroxisome proliferator-activated receptor-γ coactivator 1-β (PGC-1β), resulting in mitochondrial biogenesis defects 54; Top IIβ is required for ANTs-induced tumor protein 53 (p53) activation and apoptotic pathways, and ANTs-induced ROS production is also dependent on Top IIβ-mediated reduction of antioxidant enzyme gene transcription 54. In addition, ANTs decreased the expression of uncoupling proteins 2 and 3 that regulate mitochondrial ROS production 55. Most importantly, deletion of cardiac Top IIβ protected mice from ANTs-induced cardiomyopathy.

Notably, AIC is a disease resulting from the combined participation and crosstalk among injuries to multiple cell types, including cardiomyocytes, endothelial cells, macrophages, and fibroblasts. For instance, DOX induces DNA double-strand breaks in endothelial cells, fibroblasts, and cardiomyocytes, which may be particularly associated with signaling pathways regulated by RAC1/CDC42 56. DOX also induces persistent DNA damage and a fibrotic phenotype in vascular endothelial cells 57. Autophagy dysregulation is observed in DOX-treated endothelial cells, and restoring autophagic flux ameliorates endothelial-to-mesenchymal transition and eliminates ROS 39. In chronic AIC models, the cGAS-STING pathway is specifically activated in endothelial cells and macrophages. Endothelial cell-specific knockout of STING regulates cardiomyocyte mitochondrial bioenergetics, thereby significantly preventing the occurrence of cardiotoxicity 58.

## 2. Mitochondrial quality control in AIC

**Figure 2:**
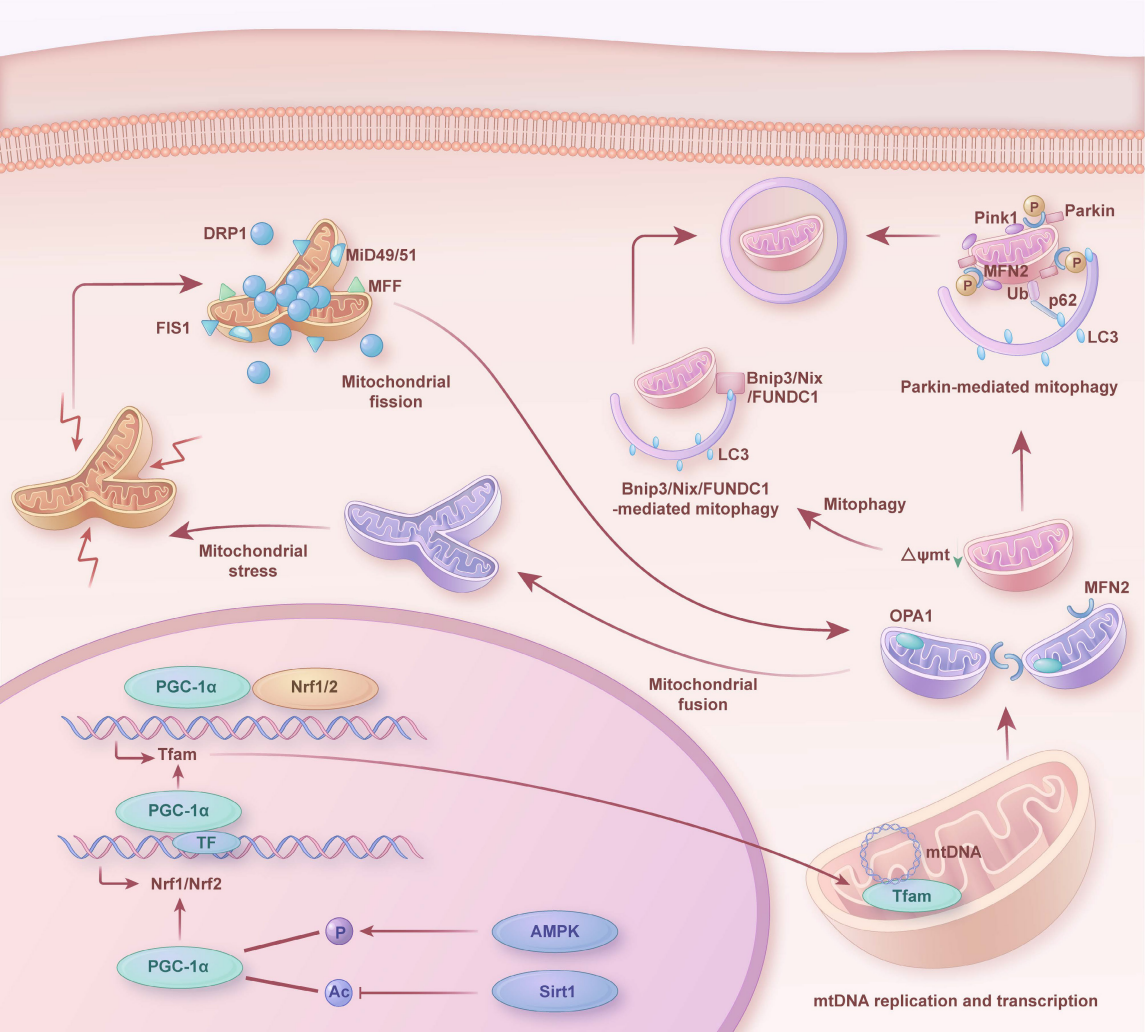
** Mitochondrial quality control mechanisms in heart.** Dynamic changes and interactions of mitochondrial biogenesis, mitophagy, mitochondrial fission and fusion in the healthy heart. Ac: acetylation; P: phosphorylation; mtDNA: mitochondrial DNA; Ub: ubiquitin.

As depicted in Figure 2, in a healthy state, mitochondria preserve their morphology, distribution, and quantity through continuous division and fusion. Abnormal mitochondria are removed by mitophagy (such as PTEN-induced kinase 1 (Pink1)/Parkinson juvenile disease protein 2 (Parkin), FUN14 domain containing 1 (FUNDC1), adenovirus E1B 19kDa interacting protein 3 (Bnip3), and NIP3-like protein X (Nix)-mediated mitophagy) to maintain cell homeostasis. Simultaneously, mitochondrial biogenesis supplies fresh components to replace damaged or senescent mitochondria. A coordinated interplay exists among mitophagy, mitochondrial biogenesis, and mitochondrial dynamics. Specifically, upon mitochondrial damage, mitochondrial fission separates damaged mitochondrial components from healthy mitochondria, producing unequal daughter mitochondria. The damaged daughter mitochondria subsequently undergo mitophagy, where they are engulfed and degraded. Conversely, healthy daughter mitochondria receive signals of high energy demand, mitochondrial fusion generates new, enlarged mitochondria. While, mitochondrial biogenesis continuously furnishes fresh components to the mitochondria, and coordinates with mitochondrial dynamics and mitophagy to uphold the renewal, quantity, morphology, and functionality of mitochondria. This intricate balance ensures the normal metabolism and survival of cells.

**Figure 3:**
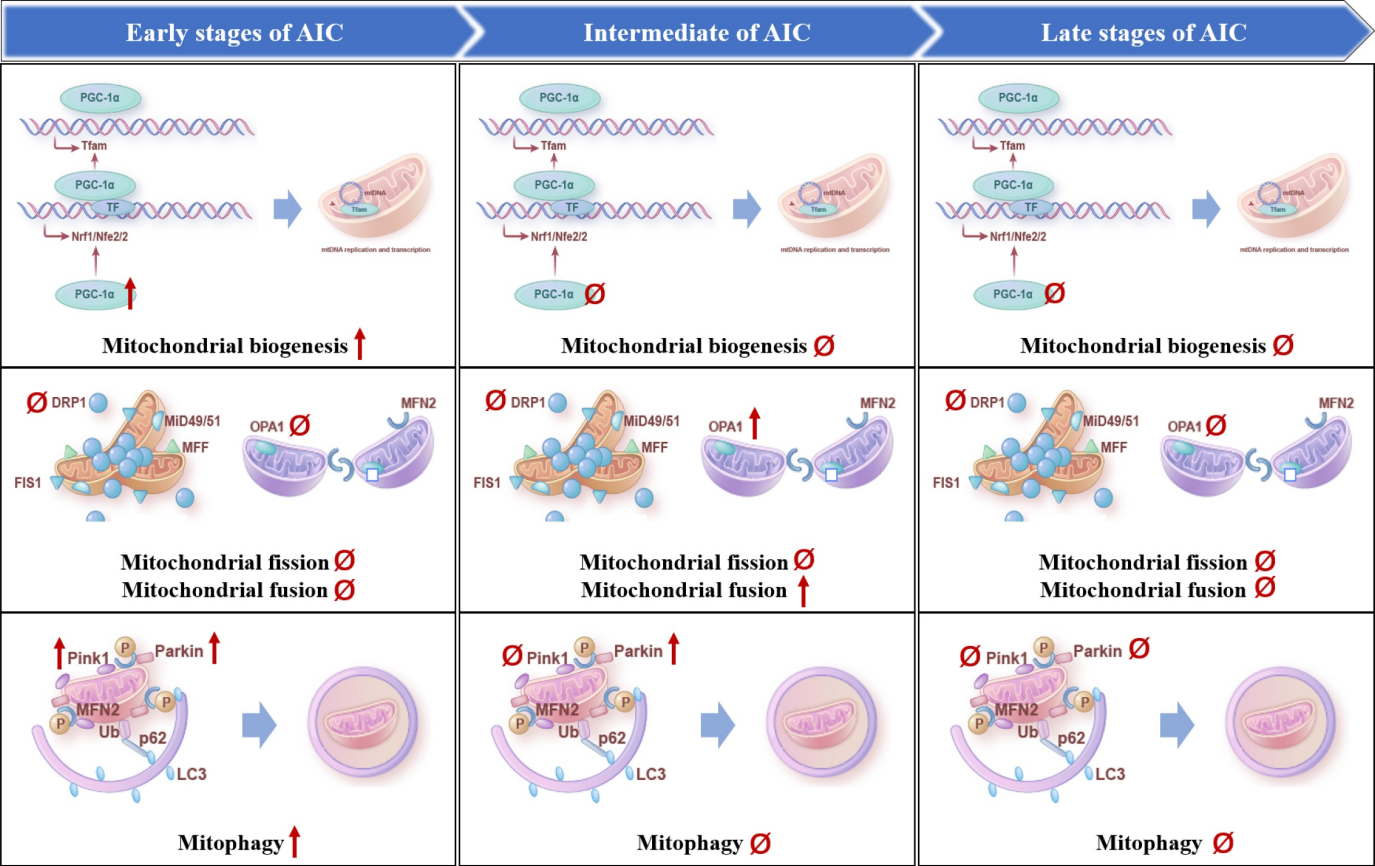
** Evolution of mitochondrial quality control mechanisms in AIC.** The temporal changes in mitochondrial biogenesis, mitophagy, mitochondrial fission and mitochondrial fusion occurred in the heart during the development of AIC, respectively, including the early stage at 1 week after DOX treatment, the middle stage at 9 weeks after DOX treatment, and the late stage at 15 weeks after DOX treatment. Upward arrows represent up-regulation, downward arrows represent down-regulation, and Ø represents no change.

**Figure 4:**
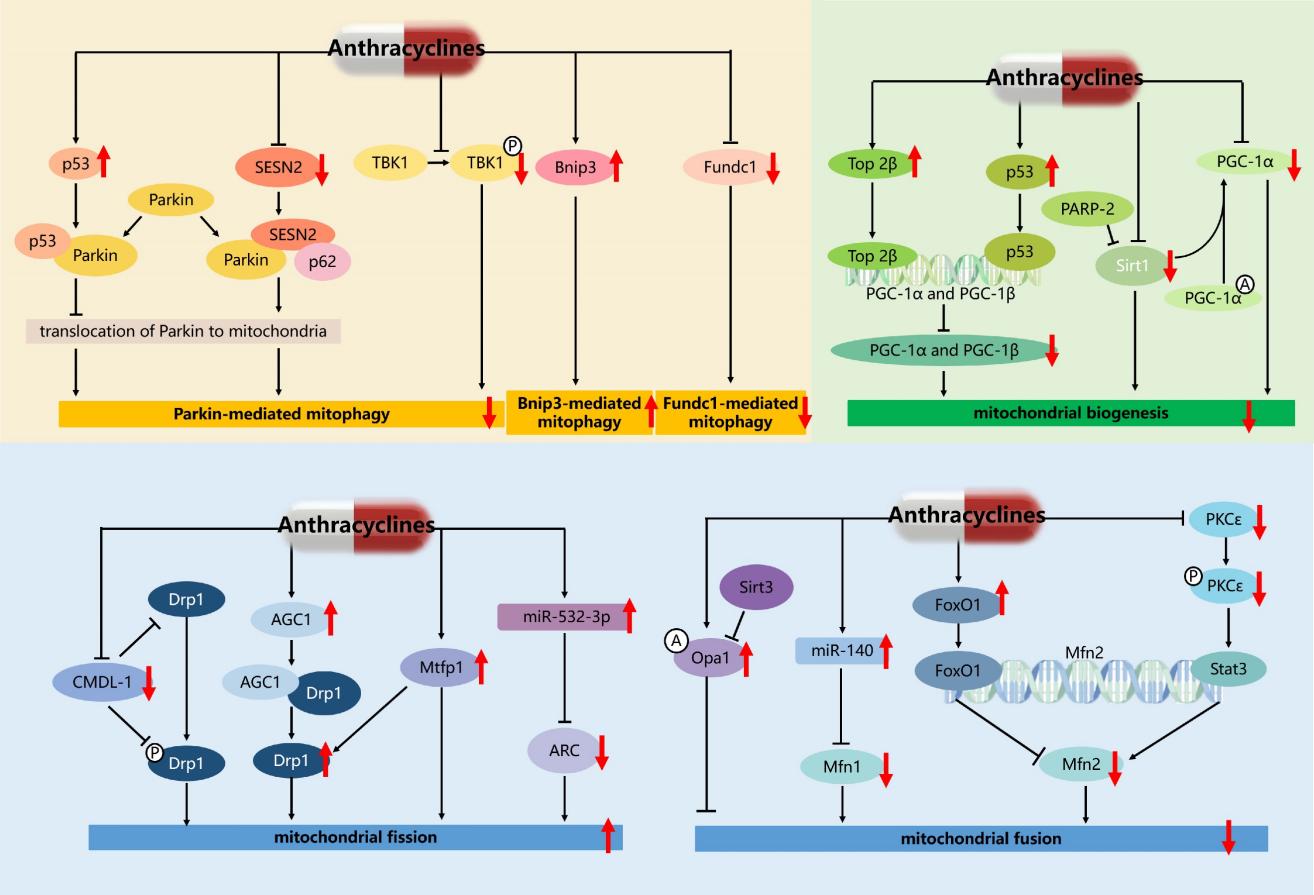
** Regulatory network of mitochondrial quality control mechanisms in AIC.** The red downward arrow represents the inhibitory effect of anthracyclines, and the red upward arrow represents the activating effect of anthracyclines. A: acetylation; P: phosphorylation.

Mitochondria are multifaceted organelles that are essential in every organ of the body and are most abundant in the heart, where they control the metabolism and survival of cardiac cells 59. Mitochondrial dysfunction has been identified as a major driver of AIC. ANTs-treated cardiomyocytes showed mitochondrial structural and functional abnormalities (such as organelle fragmentation, cristae loss and matrix destruction), and increased DNA damage and generation of ROS 60. To maintain mitochondrial integrity and function, a variety of cellular mechanisms have evolved, including processes such as mitophagy, mitochondrial dynamics (fission/fusion), mitochondrial biogenesis, and are collectively referred to as mitochondrial quality control mechanisms 61, 62. Impaired mitochondrial quality control mechanisms aggravate mitochondrial dysfunction, and recent studies have shown that ANTs-induced dysregulation of mitochondrial quality control mechanisms may be a major contributing factor to AIC 63, 64. Here, we review the research progress on dysregulated mitochondrial quality control in AIC, as shown in Figure 3 and 4.

### 2.1 Mitophagy in AIC

As shown in Figure 2, mitophagy is an organelle-specific autophagy, which is specifically used to remove damaged mitochondria and unwanted mitochondria to alleviate mitochondrial damage stress. Mitophagy has been classified into classicalPink1Parkin-mediated autophagy and microtubule associated protein 1 light chain 3 (LC3) receptor-mediated autophagy 65. Thereinto, the latter requires the involvement of several mitophagy receptors, including Bnip3, Nix, and FUNDC1.

Several studies have shown that ANTs induce the dysregulation of mitophagy in cardiomyocytes, leading to abnormal mitochondrial structure and function and cell death 66-70. Among them, activated Bnip3 by DOX triggers mitochondrial fragmentation, mitophagy, and necrosis 68, downregulated FUNDC1 by DOX blocks autophagic flux by inhibiting autophagosome biogenesis 71, and reduced or activated Parkin and Pink1 by DOX disturbs mitophagy flux 69, 70. In especial, Parkin-knockout mice showed impaired mitophagy and aggravated DOX-induced mitochondrial damage and cardiotoxicity 66, suggesting the key role of Pink1/Parkin-mediated mitophagy in AIC. However, the role of DOX on the regulation of Pink1 and Parkin remains controversial. For example, in the hearts of mice treated with DOX, Pink1 and Parkin proteins translocating on mitochondria were downregulated, and their mediated mitophagy was inhibited, resulting in dysfunctional mitochondrial accumulation and cardiomyocyte death 66. On the contrary, DOX promotes mitophagy in AC16 cardiomyocytes, as shown by increased mitochondrial translocation of Pink1 and Parkin proteins 72. The above results suggest that the changes of mitophagy and involved proteins may be related to the dosage of ANTs, the method of animal model preparation, the detection technology, and the pathological stage of AIC. Latest evidence provides the first longitudinal analysis of the temporal changes in the heart that occur during the development of AIC (1-15 weeks after completion of 5 weekly intraperitoneal injections of DOX at the dose of 5 mg/kg) in mice 73 (Figure 3). The results showed that the Pink1 and Parkin were prominently overexpressed at the early stage of AIC. PINK1 expression subsequently returned to baseline levels, whereas Parkin overexpression was sustained at week 9 and normalized at week 15 73. These results indicated that mitophagy was activated compensatively at the defensive stage at the early stage of AIC (1 week after injection), while mitophagy was gradually weakened to lower than the normal level at the middle (9 weeks after injection) and late stage (15 weeks after injection).

In addition, some studies have also reported potential therapeutic targets for the treatment of AIC by targeting mitophagy 66, 74-76 (Figure 4). For example, cytosolic p53 binds to Parkin and disturbs its translocation to damaged mitochondria and their subsequent clearance by mitophagy, and facilitates mitochondrial dysfunction and HF in AIC 66. These results raise the possibility that therapeutic activation of mitophagy by promoting the translocation of Parkin to mitochondria may ameliorate AIC. Accordingly, Sestrin2 (SESN2) interacting with Parkin and sequestosome (p62), promoted accumulation of Parkin to mitochondria and then alleviated DOX-caused inhibition of Parkin-mediated mitophagy 75. And both knockout of SESN2 by sgRNA and DOX treatment resulted in the inhibition of Parkin-mediated mitophagy, marked cardiomyocytes apoptosis, and mitochondria dysfunction. Ectopic expression of SESN2 effectively protected against DOX-induced cardiomyocyte apoptosis, mitochondrial injury, and cardiac dysfunction. Additionally, cardiomyocyte-specific TANK-blinding kinase 1 (TBK1) deletion aggravated chronic AIC via inhibition of mitophagy, denoting a pivotal role of TBK1 in DOX-induced mitochondrial injury and cardiotoxicity possibly through its phosphorylation and P62-mediated mitophagy 76. These results established SESN2, TBK1, and p53 as key players in mitophagy and provided a potential therapeutic approach to AIC.

### 2.2 Mitochondrial biogenesis in AIC

As shown in Figure 2, mitochondrial biogenesis is a complex nuclei-mitochondrial process that relies on a complex gene regulatory network 77. This process involves four major steps, including transcriptional activation of nuclear-encoded mitochondrial genes, mitochondrial translocation of the corresponding proteins, amplification of mitochondrial deoxyribonucleic acid (mtDNA), and synthesis of mitochondrial phospholipids 78. PGC-1α plays a central role in a regulatory network positively governing the transcriptional control of mitochondrial biogenesis and respiratory function 77, 79.

Substantial evidences suggest that mitochondrial biogenesis is impaired in AIC. For example, it has been reported that DOX downregulates PGC-1α and its downstream proteins, such as nuclear respiratory factor 1 (Nrf1) and mitochondrial transcription factor A (TFAM) 80, and activating mitochondrial biogenesis by PGC-1α agonist, CO/heme oxygenase (CO/HO) system or miR-23a inhibitor rescues AIC 48, 81, 82. Specifically, miR-23a expression was significantly increased by DOX concentration-dependently. Inhibition of miR-23a markedly attenuates cardiomyocyte damage by directly targeting PGC-1α or p-dynamin-related protein (Drp1), thereby inhibiting mitochondria-dependent apoptosis 82. Latest research reported that PGC-1α was significantly up-regulated at 1 week after DOX treatment (5 weekly intraperitoneal injections of DOX at the dose of 5 mg/kg), regressing to baseline expression levels at subsequent 9 and 15 week after DOX treatment 73 (Figure 3). We hypothesized that there may be a compensatory activation in PGC-1α in early AIC.

Further study found that transcriptional regulation and post-translational modifications of PGC-1α in AIC are essential for regulating mitochondrial biogenesis and function (Figure 4). In particular, DOX promoted Top 2β binding to the promoters of PGC-1α and PGC-1β to block their transcription, and deletion of Top 2β rescued DOX-induced DNA damage and mitochondrial biogenesis defects 54. DOX also induces DNA damage and telomere dysfunction leading to the activation of p53, which binds to and inhibits the promoters of PGC-1α and PGC-1β, resulting in impaired mitochondrial biogenesis 79, 83. In addition, post-translational modifications regulate PGC-1α activity as well as mitochondrial biogenesis and function. PGC-1α is directly deacetylated by sirtuin 1 (Sirt1) and activated in response to changes in energy expenditure. A recent study showed that DOX downregulates Sirt1, PGC-1α, and their downstream proteins, leading to impaired mitochondrial biogenesis and cardiac dysfunction. At the same time, the reduction of Sirt1 also leads to the increase of acetylation level and inactivation of PGC-1α, which further reduces the expression of PGC-1α downstream proteins 80. Another research has reported that depletion of poly (ADP-ribose) polymerase 2 (PARP-2) prevented DOX-induced mitochondrial dysfunction and enhanced mitochondrial biogenesis through Sirt1 activation both in aortas and aortic smooth muscle (MOVAS) cells 84. These results suggest that Top 2β/p53/Sirt1-PGC-1α signaling plays an important role in DOX-induced mitochondrial biogenesis defects.

### 2.3 Mitochondrial dynamics in AIC

Mitochondria change their size, number, density, and morphology through fission and fusion in response to the metabolic state of cardiomyocytes, and the dynamic balance between these two processes is referred to as mitochondrial dynamics 85, 86. As shown in Figure 2, mitochondrial fission not only meets the high energy demands of cardiomyocytes, but also separates dysfunctional mitochondrial parts from the mitochondrial network to maintain mitochondrial health 85. Mitochondrial fusion generates new mitochondria with a heterogeneous membrane potential and diversity of mtDNA, proteins, and metabolites 87, 88. Endogenous or exogenous lesions disrupt the balance between mitochondrial fission and fusion, with devastating consequences for cardiomyocyte function and cardiac health. Mitochondrial fission is initiated by the phosphorylation of Drp1, which is recruited to the outer mitochondrial membrane (OMM) by interacting with specific adaptor proteins mitochondrial fission protein 1 (Fis1), mitochondrial fission factor (Mff), mitochondrial dynamics proteins of 49 kDa (MiD49), and mitochondrial dynamics proteins of 51 kDa (MiD51) 89. Drp1 then undergoes guanosine triphosphate (GTP)-dependent oligomerization to form ring structures that surround and shrink the mitochondria, producing two daughter mitochondria 89. Mitochondrial fusion between OMM and inner mitochondrial membrane (IMM) is mediated by mitofusion (Mfn) 1/2 and optic atrophy type 1 (Opa1), respectively 90, 91. Mfn1/2 interacts with homologs on the OMM of the corresponding mitochondria, and Opa1 regulates IMM fusion and mitochondrial cristae remodeling and maintenance 92.

At present, the dysregulation of Drp1-mediated mitochondrial dynamics and mitophagy in cardiovascular diseases has been reported controversially. In the transverse aortic constriction (TAC) mouse heart, transient activation of mitophagy is followed by downregulation of mitophagy and mitochondrial dysfunction. However, Drp1 heterozygous knockout blocks mitophagy and exacerbates the development of mitochondrial dysfunction and HF after TAC 93. In ischemic/reperfusion heart injury, cardiomyocyte-specific knockout of Drp1 induces mitochondrial elongation, inhibits mitophagy, and leads to mitochondrial dysfunction 94. On the contrary, several studies found that DOX resulted in the downregulation of mitochondrial fusion proteins Mfn1, Mfn2, and Opa1, and the up-regulation of Drp1 95, leading to mitochondrial fragmentation 96. Mfn2 overexpression restored mitochondrial fusion and attenuated DOX-induced apoptosis, oxidative stress, and cardiac dysfunction 97. Latest research provides the first longitudinal analysis of the temporal changes in the heart that occur during the development of AIC (1-15 weeks after completion of 5 weekly intraperitoneal injections of DOX at the dose of 5 mg/kg) in mice 73 (Figure 3). The results showed that the increased mitochondrial size in the intermediate disease stage (9 week after injection) was accompanied by up-regulated expression of the mitochondrial fusion protein Opa1, followed by down-regulated expression at 15 weeks. While, no significant changes were detected at any disease stage in the expression of Drp1. These different conclusions may be attributed to the dosage of ANTs, the method of animal model preparation, the detection technology, and the pathological stage of AIC, which suggests that we should strictly control the time and dose of drug action when using drug intervention.

Some studies have reported potential regulatory targets for the treatment of AIC by targeting mitochondrial dynamics (Figure 4). For example, cardiomyocyte mitochondrial dynamic-related long non-coding RNAs 1 (CMDL-1) binding to Drp1 and phosphorylated Drp1 to prevent DOX-induced mitochondrial fission, may serve as a potential therapeutic target in AIC 98. Significantly upregulated aspartate-glutamate carrier1 (AGC1) interacts with Drp1 protein to upregulate Drp1 expression, and contributes to subsequent excessive mitochondrial fission in AIC. And AGC1 knockdown protected mice from DOX-induced cardiomyopathy by preventing mitochondrial fission, while the overexpression of AGC1 in the mouse heart led to impairment of cardiac function 99. DOX up-regulated mitochondrial fission protein 1 (Mtfp1) expression and induced a significant percentage of cells to undergo mitochondrial fission and apoptosis, and knockdown of Mtfp1 prevented cardiomyocyte from undergoing mitochondrial fission by preventing Drp1 accumulation in mitochondria 100. DOX treatment of cardiomyocytes resulted in hyperacetylation of endogenous Opa1 at lysine 926 and 931 residues, mitochondrial fragmentation, and subsequent cardiomyocyte death, which were reverted back by overexpressing cells with sirtuin 3 (Sirt3) deacetylating Opa1 and elevating its GTPase activity 101. Moreover, the suppression of Mfn2-mediated mitochondrial fusion was induced by DOX-elicited upregulation of forkhead box O1 (FoxO1), which inhibited the transcription of Mfn2 by binding to its promoter sites 97. While, cardiac-specific Mfn2 transgenic mice showed preserved mitochondrial fusion and attenuated myocardial injury upon DOX exposure 97. A recent study showed that targeting protein kinase C-ε(PKCε) and enabling its phosphorylation and activation by pharmacological intervention, activated the transcription factor signal transducer and activator of transcription 3 (Stat3) which binds to the Mfn2 promoter and up-regulates its expression, and enhanced mitochondrial fusion and cardiac function 102. Therefore, the development of new therapeutic strategies of targeted regulation of mitochondrial dynamics genes, such as Drp1, Opa1, and Mfn2, is promising for overcoming the cardiotoxicity of ANTs.

In addition, miRNA plays a role in key mitochondrial dynamic proteins (Figure 4). Increased miR-140 in DOX-treated cardiomyocytes decreased Mfn1 expression by targeting the 3'untranslational region of Mfn1, and knockdown of miR-140 inhibited mitochondrial fission 103. Enforced expression of apoptosis repressor with caspase recruitment domain (ARC) *in vivo* and *in vitro* attenuated DOX-induced cardiomyocyte mitochondrial fission, apoptosis, and cardiotoxicity 104. Furthermore, miR-532-3p was found to directly target ARC and participated in DOX-induced mitochondrial fission and apoptosis 104. Therefore, the development of new therapeutic strategies based on miR-140 and miR-532-3p is promising for overcoming the cardiotoxicity of ANTs.

## 3. Natural products regulate mitochondrial quality control to prevent AIC

It is evident that, compared to natural products, currently approved clinical drugs typically undergo meticulous research and validation processes, boasting well-defined mechanisms of action and therapeutic effects. They have undergone strict regulatory oversight and quality control, ensuring their effectiveness and safety. However, as highlighted in this article, during the course of clinical application, dexrazoxane, a currently approved medication, has incrementally exhibited adverse effects such as secondary malignancies, prompting limitations on its utilization. Consequently, further clinical evidence is necessary to substantiate its clinical effectiveness and safety. In recent years, ACEI/ARB/ARNI or β-blockers, and SGLT inhibitors have been found to have new indications for preventing and treating anthracycline-induced cardiac dysfunction, but more clinical evidence is required to conclusively establish their efficacy and long-term safety. AIC, an emerging health concern, confronts challenges such as the scarcity of currently approved therapeutic options and uncertainties regarding their effectiveness and safety. Therefore, there is an urgent need to develop more effective and safe medications. Natural products, with their unique advantages of diverse biological activities, low toxicity, and abundant resources, hold immense promise as a novel avenue for the research and development of novel pharmaceuticals aimed at preventing and treating AIC. However, compared to the established cardiovascular medications, the clinical translation of natural products encounters significant hurdles. Specifically, certain products exhibit low bioavailability, and necessitates extensive foundational research and clinical trials to affirm their efficacy and safety. This comprehensive validation process entails considerable time and financial investments. In light of the pivotal significance of mitochondrial quality control in AIC elucidated above, targeted regulation of mitophagy, mitochondrial dynamics, and mitochondrial biogenesis emerges as a promising avenue for both the prevention and therapeutic intervention of AIC. This comprehensive review endeavors to encapsulate the recent advance made in the realm of natural compounds targeting mitophagy, mitochondrial dynamics, and mitochondrial biogenesis to protect against AIC as shown in Table 2.

### 3.1 Natural products regulate mitophagy to prevent AIC

Harpagoside is the main component of the *Scrophulariae*, which exerts its salutary effects by impeding the interaction between the tumor suppressor p53 and mitophagy protein Parkin to promote mitophagy and reduce AIC 105. Similarly, aucubin, also derived from Chinese herb *Scrophulariae*, modulates autophagy and apoptosis through intricate crosstalk between nuclear factor erythroid 2-related factor 2 (NRF2) and Homeodomain interacting protein kinase 2 (HIPK2) signaling pathways to alleviate AIC 106. Luteolin is a natural product extracted from vegetables and fruits with a pleiotropic range of biological effects, including antioxidant, anti-tumor, and anti-inflammatory properties. Luteolin has been reported to attenuate AIC, including apoptosis, accumulation of ROS, and loss of mitochondrial membrane potential (MMP). Specifically, luteolin achieves these benefits by promoting mitochondrial Drp1 Ser 616 phosphorylation and upregulating transcription factor EB (TFEB) expression, thereby stimulating mitophagy 107.

### 3.2 Natural products regulate mitochondrial biogenesis to prevent AIC

Salidroside, a phenylpropanoid glycoside isolated from the medicinal plant *Rhodiola rosea*, has garnered attention for its multifaceted biological activities including anti-aging, anti-cancer, anti-inflammatory, and antioxidative functions 108, 109. A recent investigation has unveiled that salidroside significantly improved DOX-induced cardiac dysfunction, ferroptosis-like cell damage, and fibrosis. Mechanistically, salidroside suppressed DOX-triggered mitochondrial ROS generation, Fe^2+^ accumulation, and lipid peroxidation as well as restored MMP by promoting mitochondrial biogenesis, improving mitochondrial iron-sulfur clusters, and restoring mitochondrial oxidative phosphorylation (OXPHOS) complexes, thereby improving mitochondrial function 110. Cannabidiol, a major non-psychoactive phytocannabinoid in cannabis 111, is an effective treatment for some forms of epilepsy and pain 112-115. Cannabidiol has been shown to safeguard against DOX-induced cardiomyopathy by regulating mitochondrial function and biogenesis 116. Ferruginol, a diterpenoid compound, enhances cardiac function and reduces myocardial structural damage and apoptosis. Specifically, ferruginol augments the transcription factor activity of PGC-1α by enhancing the expression of deacetylase Sirt1, thereby promoting the expression of PGC-1α-mediated mitochondrial biogenesis and fatty acid oxidation genes 80. Cryptotanshinone, a key bioactive constituent extracted from *Salvia militorrhiza*, possesses various pharmacological activities, including cardiovascular protection, neuroprotection, anti-fibrosis, anti-metabolic disorder, anti-tumor, and anti-inflammatory activities 117. A study has reported that cryptotanshinone protects against DOX-induced mitochondrial dysfunction in cardiomyocytes. Mechanically, cryptotanshinone promotes ATP production, inhibits superoxide anion free radicals, and promotes the expression of mitochondrial biogenesis related factors PGC-1α, NRF-1, and TFAM 118. Collectively, these natural products, by virtue of their ability to promote mitochondrial biogenesis in the setting of AIC, effectively mitigate oxidative stress and enhance mitochondrial function in cardiomyocytes, offering promising avenues for therapeutic intervention.

### 3.3 Natural products regulate mitochondrial dynamics to prevent AIC

Liensinine, an isoquinoline alkaloid derived from plants, is a recently recognized mitophagy inhibitor that synergizes in combination with DOX for the treatment of breast cancer. It was also found that liensinine had a protective effect against DOX-induced cardiomyopathy 119. Liensinine alleviates DOX-induced cardiac dysfunction and apoptosis by reining in Drp1-orchestrated excessive mitochondrial fission 120. Paeonol is a bioactive compound extracted from the root of *Paeonia albiflora* and has a long history of clinical use. Currently, the dosage forms of paeonol approved by the Food and Drug Administration of China for human use include tablets, injections, and ointments. Oral and injection administrations of paeonol effectively quell inflammation/pain-associated ailments, ranging from rheumatoid arthritis to headaches and muscular discomfort 121. Moreover, paeonol harbors significant pharmacological promise for safeguarding cardiac and mitochondrial health 122. Indeed, paeonol enhanced Mfn2-mediated mitochondrial fusion and restored mitochondrial and cardiac function. Mechanistically, paeonol promoted Mfn2-mediated mitochondria fusion by directly targeting PKCε to activate the transcription factor Stat3, which bound to the Mfn2 promoter in a direct manner and up-regulated its transcriptional expression. In addition, paeonol did not affect the antitumor effect of DOX on a variety of tumor cell 102. Irisin, a novel exercise-mediated myokine, plays an important role in cardiovascular diseases by regulating cellular energy metabolism. Irisin combats DOX-induced oxidative stress injury in cardiomyocytes by improving mitochondrial dynamics and enhancing the endogenous antioxidant system in an adenosine 5'-monophosphate (AMP)-activated protein kinase (AMPK)-Nrf2-dependent manner, thereby protecting the heart from DOX-induced cardiotoxicity 123. Ellagic acid, a natural polyphenolic compound, targets mitochondrial fission by impeding Bnip3 to attenuate DOX-induced phosphorylation of Drp1 Ser616 68.

Similarly, luteolin, another natural polyphenol, ameliorates DOX-induced toxicity in H9c2 and AC16 cells by suppressing DOX-induced Drp1 expression and phosphorylation at Ser 616 124. Resveratrol, a natural polyphenol, was found to increase the expression of mitochondrial fusion proteins Mfn1 and Mfn2, restore mitochondrial dysfunction, and mitigate oxidative stress, thereby safeguarding against DOX-related cardiac injury 125. Honokiol, a component of the genus *Magnolia* including *M. officinalis*, *obovata*, and *grandifolia*, has been found to be an effective antioxidant and possess the antiangiogenic, anti-inflammatory, and antitumor properties 126, 127. Here, we found that honokiol promotes mitochondrial fusion by preserving Opa1 and Mfn1 levels, improves mitochondrial function, and reduces mitochondrial DNA damage 128. Collectively, these natural products accommodating mitochondrial fusion and mitochondrial fission in AIC could effectively improve cardiac and mitochondrial function in cardiomyocytes, offering promising therapeutic avenues.

## Conclusions

ANTs, as the cornerstone of chemotherapy for an array of malignancies, undeniably enhance the long-term survival of cancer patients, albeit at the expense of severe and dose-dependent cardiovascular complications, especially cardiotoxicity. The prevalence of AIC has escalated in recent years, ushered in an epidemic of cardio-oncology. This review delves into the current clinical types, incidence, and prevention and treatment strategies of AIC, and meticulously maps out the intricate molecular mechanism networks underlying AIC, in order to illuminate potential novel molecular targets for the development of prevention and treatment agents for AIC. Of particular note, mitochondrial dysfunction has been identified as a major driver of AIC. Impaired mitochondrial quality control mechanisms aggravate mitochondrial dysfunction and become a major contributing factor to AIC. Consequently, we also review the research progress of dysregulated mitochondrial quality control in AIC and natural products that specifically target these mechanisms, so as to provide potentially effective new drugs for the prevention and treatment of AIC in clinical practice.

During the meticulous review process, we discerned that the clinical manifestations corresponding to different AIC subtypes exhibit marked variability. Acute AIC mainly shows arrhythmia on the basis of preserved cardiac function, while chronic AIC shows cardiomyopathy characterized by abnormal cardiac function, often culminating in irreversible myocardial damage. Most clinical trials focus on patients with chronic AIC, and calculate the incidence of AIC and the remission rate of test drugs at different cumulative doses. We found that the incidence of AIC was significantly correlated with the cumulative dose of ANTs and the definition criteria of AIC. Therefore, the dose of ANTs should be strictly regulated during clinical use, and the changes of serum biochemical markers and cardiac function should be monitored throughout the course of chemotherapy, adhering to the principle of early detection and early treatment to reduce the incidence of cardiotoxicity in patients. Nevertheless, amidst the clinical prevention and treatment strategies for AIC, the high-cost liposomal DOX, SGLT-2 inhibitor, ACEI, ARB, ARNI, and β-blocker with insufficient clinical evidence have not been widely used, and only DXZ has been widely employed in the prevention of clinical AIC, but its concomitant side effects and effectiveness have also been questioned.

In conclusion, the effectiveness and safety of existing drugs for AIC still need to be supported by more clinical evidence, and more new drugs for the prevention and treatment of AIC are urgently needed to be developed. Furtherly, a profound understanding of the pathogenesis and drug targets of AIC is essential for advancing the development of therapeutic agents against AIC. As a regulatory mechanism to maintain mitochondrial homeostasis, the importance of mitochondrial quality control in AIC has been continuously confirmed. Among them, there are contradictory views on the activation and inhibition of mitophagy and the balance between mitochondrial fission and fusion, which may stem from disparities in the detection technology, the stage of the disease, and other confounding factors. Recent advancements have witnessed the emergence of natural products that specifically target mitochondrial quality control, demonstrating promising cardioprotective effects. But the translation of these agents into clinical practice poses major challenges, stemming from their potential adverse effects and the elusiveness of mechanisms of action, as well as the variability of mitochondrial biology in response to various stress conditions. Therefore, the focus of further research is to determine the precise treatment regimen and specific targets of action of different drugs targeting and regulating quality control mechanisms, as well as the clinical efficacy and safety of these drugs in AIC patients.

## Funding

This work was supported by Noncommunicable Chronic Diseases-National Science and Technology Major Project [grant numbers 2023ZD0502605], Beijing-tianjin-hebei Basic Research Cooperation project [grant numbers J230033], the National Natural Science Foundation of China [grant numbers 82374420, 82174364], the Science and Technology Department of Beijing University of Chinese Medicine [grant numbers 2022-JYB-JBZR-001], and Science and Technology Department of Beijing University of Chinese Medicine [grant numbers BZY-BZX-2022-08].

## Figures and Tables

**Table 1:** Effects of ANTs on cardiac mitochondrial quality control mechanisms.

Animals	ANTs treatment	Changes of mitochondrial quality control in AIC	Changes of heart or cardiomyocytes in AIC	References
Mouse	DOX was administrated in five equal intraperitoneal injections (each containing 2.5 mg▪kg^-1^) over a period of 2 weeks, and killed them 6 weeks after injection.	Pink1 and Parkin proteins translocating on mitochondria were downregulated, and their mediated mitophagy was inhibited.	Dysfunctional mitochondrial accumulation and cardiomyocyte death.	66
Postnatal rat cardiac myocytes	Treated with 10 μM DOX for 18 h.	Drp1 was phosphorylated (Drp1 616) and coincided with excessive mitochondrial fragmentation and mitophagy.	Increased necrotic cell death of cardiac myocytes.	68
Adult zebrafish and embryos	Zebrafish embryos were incubated with DOX (90 μM), and adult zebrafish were intraperitoneally injected with DOX (20 μg▪g^-1^).	Parkin activation and excessive mitophagy.	Pericardial edema, myocardial vacuolization, and apoptosis.	69
Rat	DOX was administered intraperitoneally at 5 mg▪kg^-1^ weekly for consecutive 6 weeks.	DOX disrupted Pink1/Parkin-mediated mitophagy and caused lysosomal impairment.	Aggravated mitochondrial oxidative stress, decreased antioxidant levels, and disrupted mitochondrial membrane potential (MMP).	70
Mouse	The mice were administrated with DOX at 6 mg▪kg^-1^ through the tail vein on days 0, 2, and 4.	DOX blocked autophagic flux by inhibiting autophagosome biogenesis, which was attributed to the downregulation of FUNDC1 and disruption of mitochondrial-endoplasmic reticulum contacts structures.	Cardiac remodeling and cardiac dysfunction.	71
AC16 cardiomyocytes	Treated with 0-250 nM DOX for 24 h.	Pink1/Parkin-mediated mitophagy activation and PGC-1α-mediated mitochondrial biogenesis inhibition.	Mitochondrial ultrastructural alterations, mitochondrial superoxide accumulation, and decreased MMP and mitochondrial DNA copy number.	72
Mouse	DOX was administered intraperitoneally at 5 mg▪kg^-1^ weekly for consecutive 5 weeks, followed by serial echocardiography for 15 weeks.	Pink1 and Parkin were overexpressed at the early stage of AIC. Pink1 expression subsequently returned to baseline levels, whereas Parkin overexpression was sustained at week 9 and normalized at week 15. PGC-1α was significantly upregulated at 1 week after DOX treatment, regressing to baseline at subsequent 9 and 15 week. Increased mitochondrial size in the intermediate stage (9 week) was accompanied by upregulated mitochondrial fusion protein Opa1, followed by downregulated expression at 15 weeks. While, no significant changes of Drp1 were detected at any stage.	Cardiac atrophy, reduced cardiomyocyte area, and decreased LVEF.	73
Mouse	DOX was administered at single dose (15 mg▪kg^-1^) through the tail vein.	Increased MsrB2 protein level disturbed mitophagy pathway.	Cardiac dysfunction, remodeling, fibrosis, cardiomyocyte hypertrophy, apoptosis, and oxidative stress.	74
Rat	DOX was administrated intraperitoneally by three times at 4 mg▪kg^-1^ over a period of 15 days.	DOX induced the inhibition of Parkin-mediated mitophagy by decreasing the interactions of SESN2 with Parkin and p62.	Cardiac dysfunction and cardiomyocytes injury indicated by morphological changes, disorganized myocardium, and increased cell gap, extracellular matrix, cytoplasmic vacuolization, and fibrosis.	75
Mouse	DOX was administered intraperitoneally once weekly for four weeks at 6 mg▪kg^-1^.	DOX induced mitophagy disorder, which was significantly exacerbated by Tbk1 knockdown.	DOX decreased TBK1 phosphorylation, resulting in compromised myocardial function, obvious mortality, and overt interstitial fibrosis.	76
Mouse	DOX was administered once weekly for four weeks at 5 mg▪kg^-1^ via the tail vein.	DOX inhibited the expression and activity of PGC-1α by promoting the expression of Sirt1, thereby increasing the expression of PGC-1α-regulated mitochondrial biogenesis and fatty acid oxidation genes.	Cardiac dysfunction, myocardial structural damage, and apoptosis.	80
Mouse	DOX was intraperitoneally administered at single dose (15 mg▪kg^-1^).	The mouse hearts of AIC fail to upregulate the nuclear program for mitochondrial biogenesis and its associated anti-apoptosis proteins, leading to severe mtDNA depletion.	Sarcomere destruction, apoptosis, necrosis, excessive wall stress, and fibrosis.	48
Mouse	DOX was intraperitoneally administered at single dose (25 mg▪kg^-1^), and killed them 16 h later.	Cardiomyocyte-specific deletion of Top2b protected cardiomyocytes from DOX-induced DNA double strand breaks and transcriptome changes that are responsible for defective mitochondrial biogenesis and ROS formation in AIC.	Cardiomyocyte-specific deletion of Top2b protected mice from the development of DOX-induced progressive HF.	54
Mouse	DOX was intraperitoneally administered at single dose (7.5 mg▪kg^-1^), and killed them 7 days later.	DOX induced DNA damage and telomere dysfunction leading to the activation of p53 which binds to and inhibits the promoters of PGC-1α and PGC-1β, resulting in impaired mitochondrial biogenesis.	Deteriorated cardiac systolic function.	79
Mouse	DOX was intraperitoneally administered at single dose (10 mg▪kg^-1^), and killed them 7 days later.	Decreased the expression of PGC-1α.	DCM-like phenotype with marked morphologic alteration in cardiac tissue and functional derangements.	81
Neonatal rat ventricular myocytes	Treated with 1μM DOX for 24 h.	DOX reduced PGC-1α protein and increased p-Drp1 protein, which were largely reversed by miR-23a inhibitor.	Reduced cell viability and MMP and increased cell death and ROS production.	82
Mouse	DOX was intraperitoneally administered at single dose (15 mg▪kg^-1^), and killed them 5 days later.	Decreased mtDNA copy number and expressions of key regulators for mitochondrial biogenesis.	Deteriorated cardiac systolic function.	83
Mouse	DOX was intraperitoneally administered at single dose (25 mg▪kg^-1^), and killed them 2 days later.	DOX induced mitochondrial dysfunction and mitochondrial biogenesis inhibition, which were largely reversed by the depletion of PARP-2 through Sirt1 activation.	Deteriorated vascular smooth muscle function.	84
Mouse	DOX was administered intraperitoneally at 4 mg▪kg^-1^ weekly for consecutive 3 weeks, and killed them 6 weeks after the first injection.	DOX induced Drp1 activation and mitochondrial fission through NOX1 and NOX4 activation.	DOX-induced DCM characterization disclosed that NLRP3 inflammasome activation and pyroptosis.	95
Rat	DOX was administered intraperitoneally at 2 mg▪kg^-1^ weekly for consecutive 7 weeks.	DOX decreased the expression of mitochondrial fusion proteins (Mfn1, Mfn2, Opa1) and increased the expression of mitochondrial fission proteins Drp1 and the activation of mitophagy.	Augmented mPTP susceptibility and apoptotic signaling.	96
Mouse	DOX was administered intraperitoneally at 5 mg▪kg^-1^ weekly for consecutive 3 weeks.	DOX inhibited Mfn2-mediated mitochondrial fusion.	Increased cell apoptosis and oxidative stress, and cardiac dysfunction.	97
Rat	DOX was administered intraperitoneally at 4 mg▪kg^-1^ weekly for consecutive 6 weeks.	DOX induced mitochondrial fission in cardiomyocytes.	Cardiac hypertrophy.	98
Mouse	DOX was administered intraperitoneally at 2.5 mg▪kg^-1^ three times weekly for 2 weeks, and killed them 4 weeks later.	DOX increased Drp1 Ser616 phosphorylation and mitochondrial fission.	DOX induced mitochondrial dysfunction, cardiac apoptosis and DCM.	99
HL-1 cardiac cells	Treated with 1μM DOX for 24 h.	DOX upregulated Mtfp1 expression and induced a significant percentage of cells to undergo mitochondrial fission.	Increased apoptosis of cardiac cells.	100
Neonatal rat cardiomyocytes	Treated with 1μM DOX for 24 h.	DOX treatment resulted in hyperacetylation of endogenous Opa1 and mitochondrial fragmentation, which were reverted back by overexpressing cells with Sirt3 deacetylating Opa1 and elevating its GTPase activity.	Increased death of neonatal rat cardiomyocytes.	101
Neonatal rat cardiomyocytes	Treated with 1μM DOX for 1-12 h.	Decreased Mfn1 expression and activated mitochondrial fission.	Increased apoptosis of neonatal rat cardiomyocytes.	103
Mouse	DOX was intraperitoneally administered at 4 mg▪kg^-1^ weekly for 4 weeks, and killed them 7 days later.	DOX induced cardiomyocyte mitochondrial fission.	Increased cardiomyocyte apoptosis.	104

**Table 2:** Natural products regulate mitochondrial quality control to prevent AIC.

Drugs	Animals	ANTs treatment	Effects on mitochondrial quality control	Effects on heart or cardiomyocytes	References
Harpagoside	Mouse	DOX was administered once weekly for four weeks at 5 mg▪kg^-1^ via the tail vein.	Inhibiting the binding of p53 and mitophagy protein Parkin to promote mitophagy	Improving cardiac function and reducing myocardial cell apoptosis and oxidative stress.	105
Aucubin	Mouse	DOX was administered once weekly for four weeks at 5 mg▪kg^-1^ via the tail vein.	Activating autophagy by the crosstalk between NRF2 and HIPK2.	Improving cardiac function and reducing myocardial cell apoptosis and oxidative stress.	106
Luteolin	Adult murine cardiomyocytes	1 μM DOX for 24 h.	Promoting mitochondrial Drp1 Ser616 phosphorylation and upregulates TFEB expression, thereby to activate mitophagy.	Inhibiting apoptosis, accumulation of ROS, and loss of MMP.	107
Salidroside	Mouse	DOX was administered intraperitoneally at a single dose of 10 mg▪kg^-1^.	Promoting mitochondrial biogenesis.	Inhibiting DOX-induced mitochondrial ROS, Fe^2+^, and lipid peroxidation as well as restored MMP by improving mitochondrial iron-sulfur clusters and restoring mitochondrial OXPHOS complexes thereby to improve mitochondrial function.	110
Cannabidiol	Mouse	DOX was administered intraperitoneally at a single dose of 20 mg▪kg^-1^.	Activating myocardial mitochondrial biogenesis manifested as increased mitochondrial copy number, and mRNA expression of PGC-1α, PPARα, and estrogen-related receptor alpha (ERRα).	Inhibiting myocardial injury, myocardial oxidative and nitrative stress, myocardial cell death, and cardiac dysfunction.	116
Ferruginol	Mouse	DOX was administered once weekly for four weeks at 5 mg▪kg^-1^ via the tail vein.	Enhancing the transcription factor activity of PGC-1α by promoting the expression of deacetylase Sirt1, thereby to increase the expression of PGC-1α-mediated mitochondrial biogenesis and fatty acid oxidation genes.	Enhancing cardiac function and reducing myocardial structural damage and apoptosis.	80
Cryptotanshinone	Rat	DOX was administered intraperitoneally once every 2 days for six times at 1.25 mg▪kg^-1^.	Promoting mitochondrial biogenesis-relative factors PGC-1α, NRF-1, and TFAM.	Significantly promoting the energy production of ATP by increasing the complexes activities, raising MMP, and inhibiting superoxide anion free radical.	118
Liensinine	Mouse	DOX was administered intraperitoneally at single dose of 15 mg▪kg^-1^.	Inhibiting Drp1-mediated excess mitochondrial fission, mitochondrial fragmentation, and mitophagy.	Alleviating DOX-induced oxidative stress, cytochrome C leakage, cardiomyocyte apoptosis, as well as improving mitochondrial function and cardiomyocyte contractile function.	120
Paeonol	Rat	DOX was administered intraperitoneally at the dosage of 5 mg▪kg^-1^ on days 1, 6, and 11.	Promoting Mfn2-mediated mitochondria fusion by directly targeting PKCε to activate the transcription factor Stat3, which binds to the Mfn2 promoter and up-regulated its transcriptional expression.	Restoring mitochondrial function and cardiac performance.	102
Irisin	Mouse	DOX was administered intraperitoneally once weekly for four weeks at 4 mg▪kg^-1^.	Activating AMPK-Nrf2 signaling axis to promote mitochondrial fusion.	Alleviating DOX-induced mortality, body weight loss, myocardial atrophy, damage, and oxidative stress, and cardiac remodeling and dysfunction.	123
Ellagic acid	Postnatal rat cardiac myocytes	10 μM DOX for 18 h.	Reducing mitochondria-associated Bnip3, resulting in significantly less mitochondrial fission and cell death.	Suppressing mitochondrial injury and necrotic cell death of cardiac myocytes.	68
Luteolin	H9c2 and AC16 cells	1 μM DOX for 24 h.	Decreasing mitochondrial fission protein Drp1 and Drp1 Ser616 phosphorylation.	Ameliorating DOX-induced toxicity and oxidative stress.	124
Resveratrol	Mouse	DOX was administered intraperitoneally once weekly for four weeks at 8 mg▪kg^-1^.	Increasing the expression of mitochondrial electron transport chain complexes, Mfn1, and Mfn2 in mice.	Preventing DOX-induced LV remodeling, cardiac dysfunction, and oxidative stress in mice.	125
Honokiol	Mouse	DOX was administered intraperitoneally once every 15 days for three times at 5 mg▪kg^-1^.	Promoting mitochondrial fusion.	Preventing DOX-induced ROS production, mitochondrial damage, and cell death	128
